# Non-Responsive Coeliac Disease: A Comprehensive Review from the NHS England National Centre for Refractory Coeliac Disease

**DOI:** 10.3390/nu12010216

**Published:** 2020-01-14

**Authors:** Hugo A. Penny, Elisabeth M. R. Baggus, Anupam Rej, John A. Snowden, David S. Sanders

**Affiliations:** 1 Academic Unit of Gastroenterology, University of Sheffield, Sheffield S10 2TN, UK; h.penny@sheffield.ac.uk (H.A.P.); emrbaggus1@sheffield.ac.uk (E.M.R.B.); anupam.rej@nhs.net (A.R.); 2 Lydia Becker Institute of Inflammation and Immunology, University of Manchester, Manchester M13 9PL, UK; 3 Department of Haematology, Sheffield Teaching Hospitals NHS Foundation Trust, Sheffield S10 2JF, UK; john.snowden1@nhs.net

**Keywords:** coeliac disease, non-responsiveness, refractory coeliac disease, gluten free diet

## Abstract

Coeliac disease is a common small intestinal enteropathy which manifests following ingestion of gluten in genetically susceptible individuals. Since gluten was identified as the driving factor in coeliac disease, the gluten-free diet (GFD) has remained the mainstay of treatment. While most individuals will display improvement in symptoms and signs of coeliac disease following institution of the GFD, up to 30% will continue to experience symptoms and/or have persisting intestinal inflammation. These individuals can be classified as having non-responsive coeliac disease (NRCD), which may be associated with dietary indiscretion, slow healing, refractory coeliac disease, and/or an alternative condition. The purpose of this review is to provide an overview of the causes of NRCD in adults, highlight a systematic approach to investigate these patients, and appraise the latest management aspects of this subset of coeliac disease.

## 1. Introduction

Coeliac disease (CD) is a chronic, autoimmune condition that develops in genetically susceptible individuals and has a reported prevalence of around 1% [1]. While the disease is characterized by a small intestinal enteropathy, the manifestations are broad and can involve both the gastrointestinal intestinal tract and distinct extra-intestinal sites throughout the body [2]. Inflammation and tissue damage in the small intestine results from an abnormal immune response towards ingested gluten. Persisting inflammation in active CD puts individuals at risk of osteoporosis, nutrient deficiencies, and malignancies [2]. Thus, a life-long gluten-free diet (GFD) is the mainstay treatment and reduces the long-term complications of this condition [2]. While most individuals will display improvement in symptoms and signs of CD following institution of the GFD, up to 30% will continue to experience symptoms and/or have persisting intestinal inflammation [3]. These individuals are classified as having non-responsive CD (NRCD), which may be associated with dietary indiscretion, slow healing, refractory CD (RCD), and/or an alternative diagnosis to CD [3].

In this article, we provide a comprehensive overview of the causes of NRCD in adults and highlight a systematic approach to investigate these patients. Furthermore, we appraise the latest management aspects of NRCD, with a particular focus on RCD. In doing so, it is hoped that this article will help promote the correct and timely identification, multi-disciplinary appraisal, and treatment of individuals with NRCD, in order to reduce morbidity and mortality associated with this subset of CD.

## 2. Defining Non-Responsive Coeliac Disease

It is generally accepted that NRCD is defined as persistent symptoms, signs, laboratory abnormalities, or histological changes typical of CD, despite at least 6 to 12 months of presumed adherence to a GFD [4]. Whilst this time length is often quoted in the definition of NRCD, it is arbitrary, as the length of time needed to respond to a GFD is variable and thus it is important to be guided by the clinical picture [5]. NRCD can be categorized as primary, where there is no response to a GFD, or secondary, where individuals initially respond to a GFD, but then develop symptoms despite ongoing adherence to a GFD [3]. Some expert gastroenterologists have suggested that the definition of NRCD causes confusion, primarily because the underlying cause of symptoms in these individuals may be related, or unrelated, to CD [6]. In contrast, we consider this definition appropriate, as it puts CD at the forefront of the clinical approach when considering the causes of persisting symptoms in these patients.

## 3. Causes of Non-Responsive Coeliac Disease

### 3.1. An Alternative Primary Diagnosis

An obvious but crucial first step in considering the cause of NRCD is to re-examine the primary diagnosis of CD. This is particularly important in the setting of a patient with a historical diagnosis of CD presenting with secondary non-responsiveness, because diagnostic testing in CD has advanced greatly over the last two decades, alongside a shift in our understanding of how CD manifests in adults, with a greater emphasis on extra-intestinal signs and symptoms than previously thought [2]. Indeed, case series suggest that around 8% of patients evaluated with a presumed diagnosis of NRCD did not have an original diagnosis of CD [7,8].

Confirming the diagnosis of CD is achieved by reviewing serology and histology taken at the time of diagnosis. Notably, the detection of circulating anti-gliadin antibodies (AGA) was heavily relied upon in the serological diagnosis of CD up until around the early 2000s [9]. However, it has since been demonstrated that the AGA test has poor sensitivity and specificity for CD, and this assay has now been superseded by those that detect anti-tissue transglutaminase (TTG) and -endomysial (EMA) antibodies, which have greater disease specificity [10]. In addition, selective immunoglobulin (Ig) A deficiency is 10-fold more common in patients with coeliac disease than the general population (2% vs. 0.2%, respectively) [11]. Most first-line TTG and EMA assays detect IgA antibodies. In view of this, IgA deficiency is important to test for, and, if present, IgG-based serological testing should be undertaken [12].

It is also of note that the recent past has seen the recognition of a broader group of disorders that fall under the spectrum of CD, which include ‘potential’ and ‘seronegative’ CD [2]. Whilst villous atrophy in the context of negative serology maybe due to CD, other diagnoses must be considered first, as CD only accounts for 30% of serology negative villous atrophy [13]. Other causes include immune-mediated (e.g., autoimmune enteropathy), inflammatory (e.g., eosinophilic gastroenteritis), infectious (e.g., giardiasis), iatrogenic (e.g., NSAIDs), and idiopathic causes [14]. Finally, variability exists in the identification of the main histological changes of CD, which can lead to diagnostic uncertainty [15,16].

Therefore, if re-examining the primary diagnosis raises any doubt, up to date coeliac serology and duodenal biopsies should be performed. It is noteworthy that these individuals are likely excluding gluten from their diet, so biopsies should be performed following a gluten challenge—at least 6 weeks of 10g of gluten/day [12]. In addition, human leucocyte antigen (HLA) typing can be used as a negative predictive test to exclude CD, as positive HLA typing occurs in up to 99.7% of individuals with CD [17]. However, HLA typing cannot be used to solely diagnose CD, as it is present in up to 40% of the general population [18].

### 3.2. An Associated Condition

Coeliac disease is associated with a number of conditions, including microscopic colitis, pancreatic insufficiency, small intestinal bacterial overgrowth (SIBO), inflammatory bowel disease (IBD), and lactose or fructose intolerance (Table 1) [5,19,20,21,22]. These are either related (SIBO, lactose/fructose intolerance) or unrelated (IBD, microscopic colitis) to mucosal damage in CD and can co-present at the time of index CD diagnosis or manifest after diagnosis. Therefore, the presence of these conditions should be considered as a cause for persisting symptoms in coeliac patients and managed accordingly.

In addition, functional gastrointestinal disorders, such as irritable bowel syndrome (IBS) and gastrointestinal dysmotility, are more prevalent in individuals with CD [23,24] and should not be overlooked as a cause of persisting symptoms in individuals with normal repeat duodenal histology. For these individuals, a low fermentable oligo-, di-, mono-saccharide and polyol (FODMAP) diet may improve symptoms and quality of life [25,26]. In one study, 41 CD patients with IBS who had been on a GFD for at least 1 year demonstrated significant improvement in IBS-symptom severity scores after 3 months on the low FODMAP diet [25]. The benefits of a low FODMAP diet have also been shown in a recent randomized controlled trial; 50 patients with CD and persisting symptoms were randomized to follow either a regular GFD (R-GFD, *n* = 25) or a low FODMAP GFD (LF-GFD, *n* = 25) for 21 days. At the end of the 21 days, symptoms were reduced in the LF-GFD group, but not in the R-GFD group. General wellbeing increased in both groups, but a significantly higher improvement was noted in the LF-GFD group [26]. This suggests an additive effect of combining a low FODMAP diet with a GFD in certain individuals with NRCD. However, notably, these studies did not rule-out persistent villous atrophy as a cause for ongoing symptoms. 

In addition to the low FODMAP diet, probiotics have shown potential benefits in individuals with CD and IBS symptoms. A recent randomized, double-blind, placebo-controlled multicenter trial investigated the use of a probiotic mixture in patients with CD with persisting IBS-type symptoms despite a strict GFD [27]. In total, 109 CD patients were randomized to receive either probiotics or a placebo for 6 weeks. The investigators noted a significantly greater reduction in symptoms with the use of probiotics compared with the placebo [27]. However, while these results are promising, further studies are needed to evaluate the use of the low FODMAP diet and/or probiotics in NRCD associated with functional gastrointestinal disorders.

### 3.3. Dietary Indiscretion

Ongoing gluten ingestion (either deliberate or inadvertent) is reported in around 35–50% of cases of NRCD and thus is one of the commonest causes of persistent CD [7,8]. While it has been estimated that effective adherence to a GFD occurs in only 40–90% of cases, complete non-adherence is uncommon, with most studies reporting it in less than 5% of individuals [5,28]. This underscores the difficulty in maintaining a strict GFD. Indeed, inadvertent exposure may even occur in the setting of presumed gluten abstinence, as highlighted in a recent study which reported that gluten was detected by a commercially available home testing kit in 32% of gluten-free labelled restaurant food [29].

Assessing adherence to a GFD is notoriously difficult. A detailed dietary history including the use of food diaries is an effective and commonly used method and can identify inadvertent gluten exposure, particularly if individuals lack an understanding of which foodstuffs/products contain gluten. However, as mentioned, gluten exposure can occur in the setting of presumed abstinence and thus would not be identified by this method. As gluten exposure causes on-going symptoms in patients with CD, detailed symptom assessment at follow-up is important to determine. However, gluten exposure may not lead to symptoms in all patients, and around 20% of individuals with CD are asymptomatic at diagnosis [30]. Serological markers (anti-TTG and -EMA antibodies) have traditionally been used in clinical practice to monitor for adherence. The normalization of circulating TTG titers after institution of a GFD is often (mis-)taken to reflect a reduction in inflammation and mucosal healing, which itself is considered evidence of effective adherence to a GFD. However, a recent meta-analysis interrogating the diagnostic accuracy of elevated anti-TTG and -EMA IgA antibodies for predicting persistent villous atrophy in individuals on a GFD demonstrated a specificity of 0.83 (95% confidence interval [CI] 0.79–0.87) and 0.91 (95% CI 0.87–0.94) and a sensitivity of 0.5 (95% CI 0.41–0.60) and 0.45 (95% CI 0.34–0.57), respectively [31]. Therefore, these serological tests cannot be relied upon to inform on mucosal healing after the institution of a GFD. 

In view of this, repeat duodenal biopsy is currently the best way to assess for mucosal healing and thus, indirectly inform on effective gluten abstinence [4]. However, it is difficult to predict the most appropriate time to perform repeat duodenal biopsies in CD, because the rate of mucosal recovery following the institution of a GFD varies between individuals. Some studies suggest that histological remission occurs in most individuals (68%) within the first year following diagnosis [32]. However, other observational studies have reported histological remission in 34–65% of individuals up to two years post-diagnosis, and others have suggested that mucosal recovery may even take as long as 5 years in some individuals [33,34,35,36]. It is therefore important to be aware that some patients with CD may be ‘slow responders’ to the GFD and, as such, persisting villous atrophy on follow-up biopsies may not reflect true dietary indiscretion.

The use of faecal and urine gluten immunogenic peptides (GIPs) may provide a practical non-invasive future approach to monitor for gluten exposure in individuals. In a recent study, patients with CD who reported adherence to a strict GFD had a positive GIP, despite the majority of these individuals being asymptomatic [37]. This highlights the potential utility of using GIPs in assessing ongoing gluten exposure. However, so far, these tests have failed to gain widespread acceptance into clinical practice owing to certain limitations. Namely, urine and faecal GIPs are only able to detect gluten ingestion 1–2 days and 2–4 days, prior to testing, respectively [38]. Therefore, it is possible that the window of gluten exposure is missed by the time individuals are tested for GIPs. Notably, this is not the only non-invasive marker of gluten exposure available, and the last decade has seen a rise in the development of other commercially available tests, including point of care tests [39]. However, robustly designed studies are awaited to assess their place in clinical practice.

### 3.4. Gluten Super-Sensitivity

In the UK, foods that contain 20 parts per million or less gluten can be branded as gluten free. It is generally considered that the vast majority of individuals with CD will tolerate foodstuffs with gluten at this level [5]. However, some individuals are sensitive to small traces of gluten (less than 20 parts per million) and thus display an incomplete response to a strict GFD [40].

The Gluten Contamination Elimination Diet (GCED) comprises whole, unprocessed foods and has been developed to prevent ingestion of trace amounts of gluten [40]. This has been shown to result in an improvement in symptoms in this (super-sensitive) subset of individuals with NRCD who are adherent to a GFD [40]. It is thought that the GCED may also be used to distinguish between individuals who are ‘supersensitive’ to a GFD and those with true RCD [41]. This could be particularly useful in preventing patients receiving unnecessary corticosteroid and immunosuppressive treatment for an incorrect diagnosis of RCD [4]. Notably, other diets exclusive of trace gluten have also been trialed in this setting, including the elemental diet. This has demonstrated potential benefits, with both histological and clinical improvement being demonstrated in small case series [42,43]. However, there is a lack of large, long-term studies assessing the efficacy and acceptability of either diets in this patient cohort.

### 3.5. Refractory Coeliac Disease

#### 3.5.1. Overview

RCD is defined as persistent or recurrent malabsorptive symptoms/signs with villous atrophy, despite adherence to a strict GFD for at least 12 months [44]. However, RCD may be suspected before this as patients with RCD often show little response to a GFD, meaning these patients may present with severe and/or progressive symptoms before this 12-month GFD trial period [4].

RCD has a reported prevalence of 0.3–4% of patients with CD [45,46,47,48] and is reported to account for 8–23% of NRCD cases [7,8,47]. This wide prevalence range likely reflects the difficulty in differentiating between inadvertent gluten exposure, slow responders, individuals with super-sensitivity and true RCD. As a result, RCD may be over-diagnosed and the true prevalence in this cohort is likely to be lower than that reported.

Individuals with RCD can be sub-classified as having either RCD Type 1 (RCD1) or Type 2 (RCD2), based on the abnormal expansion of a subset of small intestinal intraepithelial lymphocytes (IELs), which occurs in RCD2 [49]. RCD2 is predominantly diagnosed in adults aged 50 or above, although younger cases have been observed [48]. Patients with RCD2 are more likely to present with constitutional symptoms, such as weight loss and nutrient deficiencies, than those with RCD1, which reflects prolonged severe mucosal inflammation in these patients [46]. The presence of other symptoms, such as gastrointestinal bleeding, fever, night sweats, and bowel obstruction, should lead to the consideration of complications associated with RCD, such as enteropathy associated T-cell lymphoma (EATL) and ulcerative jejunitis (UJ) [50]. The latter, in particular, is common in patients with RCD2 and is characterized by areas of multiple chronic ulcers with a benign appearance [46].

Patients with RCD1 often show good response to treatment and the 5-year survival is 80–100% [45,46]. In contrast, treatment options are limited in RCD2 and the 5-year prognosis is around 50% [45,46,47,51,52]. Mortality in RCD2 may occur either as a result of malabsorption and consequent malnutrition, or from the development of associated complications, including EATL [45,46]. Progression to EATL is reported to occur in 33–67% of RCD2 cases; EATL itself has a 5-year survival of only around 10% [45,46,51,52].

#### 3.5.2. Diagnosis

**Figure 1:**
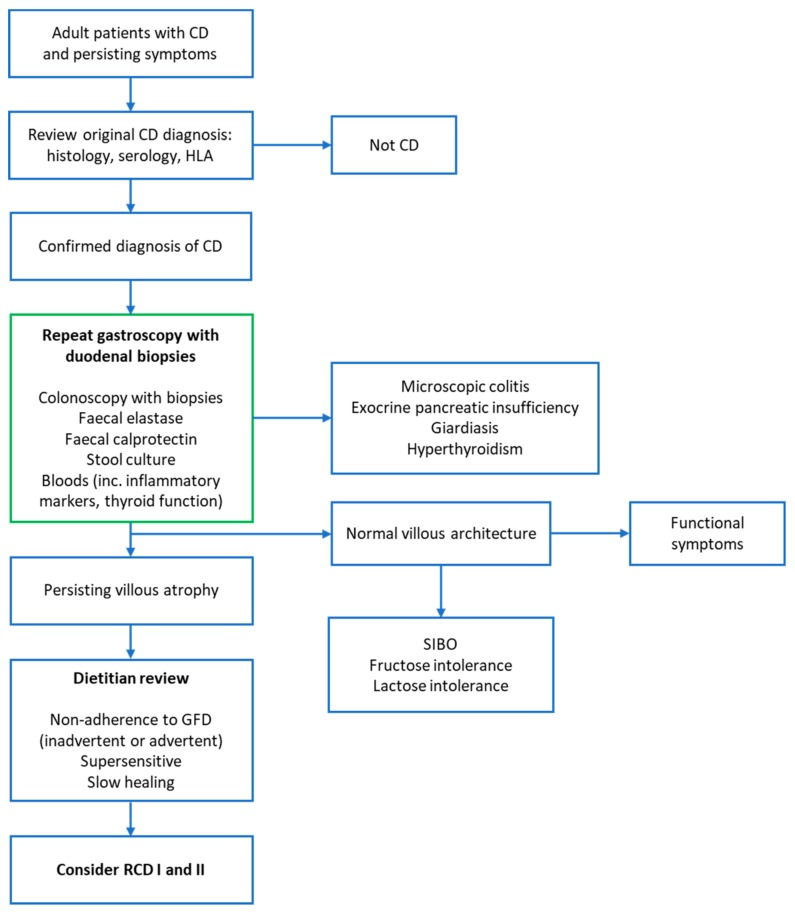
Algorithm for investigating coeliac patients with persisting symptoms. Investigations outlined in the green box can be planned for during the initial follow-up appointment in suspected non-responsive coeliac disease (NRCD). This may expedite the identification and diagnosis of RCD. Where there is clear clinical concern of ongoing gluten ingestion, a dietary review earlier in the investigation pathway may prevent unnecessary tests for some patients. However, we would always advocate repeat duodenal biopsies in individuals presenting with NRCD. Modified with permission from [4].

Persisting/worsening symptoms and villous atrophy despite strict adherence to a GFD should prompt the clinician to consider a diagnosis of RCD. A flow chart outlining the stepwise investigation of these patients is shown in Figure 1. Once RCD is suspected, detailed evaluation of up-to-date duodenal biopsies should be performed. This is beyond the scope of histological assessment of duodenal biopsies for CD diagnosis and should be undertaken at specialist centers that are experienced in the diagnosis of RCD.

Individuals with RCD2 demonstrate an abnormal expansion of a subset of small intestinal IELs [49]. These IELs are often described as being ‘aberrant’, as they lack the usual expression of cell surface proteins, such as CD3 and CD8, but do express intracellular CD3 [53,54]. It is important to note that this IEL subset is present in normal individuals, uncomplicated CD, and RCD1, but at lower frequencies to that is found in RCD2 [53,54]. In RCD2, there is evidence that aberrant IELs display clonal rearrangement of their T cell receptor (TCR) [53,54,55]. However, clonal rearrangement of the TCR is not unique to RCD2 and has been described transiently in uncomplicated CD at the time of diagnosis and in individuals with CD who continue to ingest gluten [56,57]. TCR clonality alone is therefore not an adequate indicator of RCD2, which requires quantification of the frequency of the aberrant IEL subset within the small intestine [58].

The frequency of aberrant IELs within the small intestine can be determined by either flow cytometry or immunohistochemistry (IHC). Flow cytometry is a highly specialized laser-based technique which can measure morphometric and phenotypic characteristics of individual cells simultaneously [59]. IHC is a widely available microscopy-based technique that enables visualization of cellular components by the operator. Using flow cytometry, RCD2 is diagnosed when the frequency of aberrant IELs exceeds 20% of the isolated lymphocyte pool [58]. By IHC, the threshold frequency of aberrant IELs required for a diagnosis of RCD2 is raised to >40% [58]. This is because flow cytometry is considered a more accurate method to enumerate the frequency of aberrant IELs than IHC and therefore is currently the preferred approach in RCD [60]. However, flow cytometry is subject to laborious sample preparation and variable cell yields from tissue preparations [59,60]. Furthermore, flow cytometry is not widely available, with its use being restricted to specialized centers, and further validation is necessary before wider testing can be provided. This could lead to delays in diagnosis and initiation of treatment of patients with RCD2. Notably, the cell surface receptor, NKp46, is detected on aberrant IELs [54]. Cheminant et al. [61] recently reported that the presence of >25 NKp46+ IELs/100 epithelial cells accurately discriminated RCD2 from CD and RCD1. In this study, NKp46 expression was detected by IHC staining of duodenal biopsy sections [61]. Therefore, this approach may represent an accurate, yet more widely available, alternative to flow cytometry for risk stratification/identification of individuals with suspected RCD2.

The requirement for multiple diagnostic modalities in the evolving methodology of diagnosing RCD supports the diagnosis of RCD within integrated haemato-pathology systems [62]. Currently, such specialized testing is limited to specialist centers with an interest in RCD, and further research is warranted to validate diagnostic methodology, classification criteria, and prognostic systems, as well as developing novel biomarkers before diagnostics in RCD can be delivered in a more widely available and quality assured manner.

#### 3.5.3. Management

(i) General Measures

Management of RCD is challenging, and patients should be referred to a tertiary center with experience in RCD for treatment. Treatment widely depends on the type of RCD, but general measures include nutritional support and managing the complications associated with prolonged malabsorption and malnutrition [4]. Routine investigations, including haematinics, iron studies, electrolytes, albumin, magnesium, and calcium, should be checked and replaced where necessary [4]. In individuals presenting with severe malabsorption and weight loss, trace elements, including zinc and copper, should also be checked and corrected [4]. Consideration should be taken to monitor for re-feeding syndrome alongside early nutritional support.

(ii) RCD1

The aim of management of RCD1 is to improve symptoms and promote histological recovery. Follow-up is important to assess for progression to RCD2 and/or malignancy. In a subset of patients, maintenance of a strict GFD alongside nutritional support has been demonstrated to induce symptomatic and histological improvement [45,46]. However, the mainstay of therapy is nutritional support and corticosteroids +/− azathioprine, and most patients with RCD1 achieve clinical remission and mucosal healing with this approach [50,63].

Individuals with RCD1 should be started on oral steroids (prednisolone, 0.5–1 mg/kg/day) alongside agents to prevent loss of bone mineral density [4]. The majority of patients with RCD1 will be steroid dependent [45,64]. In view of this, budesonide (9 mg/day) has been used as an alternative to prednisolone therapy, with the intention of reducing the risks associated with systemic oral steroid therapy [4]. Modified release budesonide has a systemic bioavailability of 12% [65], although it is thought that availability may be increased in those with mucosal disease. Therefore, there is the possibility that patients taking budesonide may be exposed to similar long-term risks as with oral prednisolone therapy [60]. When considering any preparation of budesonide (Entocort or Budenofalk), it is important to note that these are only bioavailable in the small bowel if the tablet is ground down or the capsule is opened [4]. Therefore, patients should be asked to grind the medication with their teeth prior to ingestion in order to optimize small bowel efficacy [66]. 

After induction of clinical remission, azathioprine can be added (2–2.5 mg/kg/day) [4]. Combination therapy, using azathioprine and prednisolone, may be superior to steroids alone, although normalization of villi is seen in only around half of patients [45,67] and there is a lack of adequately controlled studies comparing mono- or combination therapy in RCD1. There have been concerns that the use of azathioprine may increase the risk for development of lymphoma. However, a study in 43 RCD1 patients failed to demonstrate this over a follow-up period of 72 months [51]. 

Azathioprine is metabolized by the body into the pharmacologically active metabolite, thioguanine nucleotide (TGN) [68]. In individuals who fail to respond to azathioprine, TGN levels should be measured to ensure adequate drug metabolism and to enable dosage optimization [60]. A thiopurine derivative, thioguanine, has been used in a small cohort (*n* = 12) of individuals with RCD1 and demonstrated histological improvement in around 2/3rds of individuals, as well as reduced steroid dependency [68]. Infliximab may be a therapeutic option for individuals who fail to respond to azathioprine therapy; however, the evidence for its potential efficacy has only been described in case reports to date [69,70]. The benefit of small intestinal release mesalamine (SIRM) in RCD1 patients as a steroid-sparing agent has also been reported in a recent small study, although there is a lack of other data supporting these findings [71].

In individuals with RCD1, a follow-up intestinal biopsy after 3 months of treatment should be performed to assess for histological response [4]. Once treatment response has been established, it is recommended that repeat duodenal biopsies are performed annually to monitor for expansion of the aberrant IEL subset, which would suggest progression to RCD2 [4]. The withdrawal of azathioprine after 2–3 years of complete response should be considered in order to confirm the diagnosis of RCD1 rather than a slow response to gluten withdrawal [60].

(iii) RCD2

Once RCD2 is diagnosed, individuals should be assessed for EATL. Investigations to be considered should include cross-sectional imaging such as an abdominal CT scan, MR enteroclysis, a fluorodeoxyglucose-positron emission tomography scan, enteroscopy, and capsule endoscopy [72,73,74,75,76,77,78]. Following this, the management of RCD2 is aimed at symptom improvement and reducing the risk of progression to EATL. Since RCD2 is rare, the evidence base for its management is limited to a number of case series, and, notably, there is no evidence that EATL can be prevented, although treatment may delay its onset [4].

It is recommended that treatment for RCD2 is initiated with prednisolone or budesonide [4]. In one study, 77% of RCD2 patients treated with corticosteroids (*n* = 30) had a clinical response (defined as 50% reduction in symptoms and/or 50% regain of lost weight prior to treatment) and 33% showed histological improvement (7 partial improvement and 3 complete mucosal healing) with systemic corticosteroids [46]. Budesonide has also been used with some success in RCD2. In one study, 7/ 13 RCD2 patients treated with open-capsule budesonide showed a reduction in the frequency of aberrant IELs on follow-up biopsies [66] and other studies support the use of budesonide in RCD2 [64]. 

Unlike in RCD1, azathioprine is not recommended as a therapy in RCD2 due to concerns regarding lymphomagenesis with thiopurine treatment and the increased risk of EATL development in these individuals [67]. This is despite early studies showing encouraging subjective clinical and histological responses in RCD2 [67,71]. The use of the adenosine nucleoside analogue cladribine has been documented in patients with RCD2. One study of 32 patients reported clinical, histological, and immunological improvement in 81%, 47%, and 41% of patients, respectively, with progression into EATL reported in 16% of individuals [79]. Another study of 17 patients demonstrated clinical and histological improvement, and a significant decrease in the frequency of aberrant IELs in 36%, 59%, and 35% of patients, respectively [80]. However, despite mucosal recovery and a reduction in the proportion of aberrant IELs, all patients still fell within the classification for RCD2, and 41% of this group developed EATL [80]. Therefore, treatment with cladribine in RCD2 has been shown to be well tolerated and can induce clinical and histologic improvement in some patients, although it does not prevent EATL development [4].

There are a number of other immunosuppressive medications which have been trialed in small case series/ reports in RCD2, including infliximab [68,69,81], campath (anti-CD52) [46,82], methotrexate [46], ciclosporin [46,83], and recombinant interleukin (IL)-10 [84]. Recently, anti-IL-15 monoclonal antibody treatment was used in a randomized double-blind placebo-controlled study. In patients with RCD2 who were treated with an IL-15 monoclonal antibody or placebo for 10 weeks, there was no difference in the primary endpoint of reduction of aberrant IEL frequency from baseline between groups [85]. However, an improvement in symptoms was noted, and anti-IL-15 is potentially available as part of a compassionate-use programme in RCD2 [4].

Immunoablative chemotherapy, followed by autologous haemopoietic stem cell transplantation (aHSCT), now indicated in a range of autoimmune and inflammatory diseases [86,87,88], has been used rarely to treat RCD2, mainly in a single center, with limited evidence that it may prevent progression to EATL. Results of a step-up program, incorporating the use of aHSCT in patients who do not achieve histological responses to cladribine, have been published [89,90,91]. Further observational and prospective studies with greater numbers are warranted to define the risk:benefit ratio of aHSCT (and its intrinsic toxicities) and where it should feature in the treatment algorithm for RCD2. Until then, current recommendations are that aHSCT can be considered as an option for individual patients after careful discussions of risks and benefits and can be performed in accredited specialist centers with major experience and appropriate infrastructure [88].

A case report has also documented the potential benefit of faecal microbial transplant in RCD2 [92]. A patient with RCD2 who received a faecal microbial transplant for recurrent Clostridium difficile infection subsequently showed full mucosal recovery and disappearance of coeliac symptoms. Dysbiosis has been demonstrated in both CD and RCD [93,94,95,96,97,98,99,100], and collectively these studies suggest that targeting the intestinal microbiota may provide future therapeutic strategies for the treatment of RCD, although it is first necessary to understand clearly the pathological mechanisms underlying dysbiosis and CD/RCD.

## 4. Conclusions

Up to a third of individuals with CD develop NRCD. Dietary indiscretion is the commonest cause of NRCD, yet currently there is no reliable objective assessment of ongoing gluten ingestion in these patients. The diagnosis and management of RCD is challenging, and patients should be referred to a specialist center with multi-disciplinary experience in RCD for assessment, diagnostics, treatment, and follow-up. Novel therapeutic strategies are required to provide realistic treatment options in RCD2 to impact the dismal mortality in this condition.

## Figures and Tables

**Table 1:** Conditions associated with coeliac disease that should be considered as a cause for persisting symptoms in coeliac patients [5,19,20,21,22].

Pancreatic insufficiency
Inflammatory bowel disease
Lactose and/or fructose intolerance
Small intestinal bacterial overgrowth
Microscopic colitis
Irritable bowel syndrome
Functional dysmotility
